# Habitual visual acuity and visual acuity threshold demands in Nigerian school classrooms

**DOI:** 10.1038/s41598-022-21048-z

**Published:** 2022-10-24

**Authors:** Onyekachukwu Mary-Anne Amiebenomo, Ejitu Mfon Isong, Mark Eghaghe Edosa, Joy Margaret Woodhouse

**Affiliations:** 1 Department of Optometry, Faculty of Life Sciences, University of Benin, Benin City, Nigeria; 2 School of Optometry and Vision Sciences, College of Biomedical Sciences, Cardiff University, Cardiff, United Kingdom; 3 Ministry of Health, Uyo, Akwa Ibom State Nigeria

**Keywords:** Health care, Optics and photonics, Paediatric research

## Abstract

This study was designed to estimate the burden of blindness and vision impairment in school children, and to determine the proportion of students meeting the visual acuity (VA) demand for their classrooms. From 148 primary and secondary school classrooms in Edo State, Nigeria, the habitual distance and near VA of over 2000 students were measured. Values obtained were compared with the calculated distance VA demand and actual near VA demand for their classrooms. Measures used to calculate the VA demand were working distance and smallest text size on learning materials, at both distance and near. We also determined the relationship between age and calculated VA demand and the effect of factors such as school ownership and school type on the VA  demand. Habitual distance vision impairment was found in 0.8% of pupils, including 2 (0.10%) who met the WHO criteria for blindness. The average VA demand at the furthest and nearest sitting position to the board was 0.21 ± 0.23 and 0.65 ± 0.33LogMAR, respectively. Near working distance of students ranged from 15.00 to 37.20 cm, and the near VA demand as well as actual near VA demand (using a 2.5 times acuity reserve) was 0.60 ± 0.17 and 0.20 ± 1.7LogMAR, respectively. LogMAR VA demand increased (size of print decreases) with increasing age, at both distance (r =  − 0.549, *p* = 0.070) and near (r =  − 0.921, *p* < 0.0001). The VA demand at maximum distance and the actual near VA demand differed significantly by school ownership and between primary and secondary schools. Most students had VA better, but up to 11% of students per class had VA poorer than their classroom demands. Although the majority of students had better VA than their classroom demands, for students with reduced vision, learning could be negatively impacted. It is important to continually screen students for vision impairment and ensure prompt referral and treatment. These findings have implications for managing vision problems in children, as well as enabling appropriate classroom arrangements for those with vision impairment.

## Introduction

Vision is the ability to view our surroundings and make meaning of what we see. In a classroom, the ability to visualise learning materials is closely related to academic performance. Visual acuity (VA) measurement, though not a precise indicator of causes of vision defects^1^ is useful to determine the level of vision of an individual, hence, the need for vision screening and school based visual checks before and during school years, which is necessary to determine who may require comprehensive eye examination to enable optimal learning.

In many developed regions of the world, eye examinations are offered at certain ages. For example this may be within 72 h of birth, between six and eight weeks, once again at one year or between two to three years, and on entering school^2^. This is, however, not the case in Nigeria, where nationally accepted guidelines for screening children at set ages are lacking. This, therefore, means that on entering school, a child’s level of vision may be unknown. Previous studies have shown a prevalence of vision impairment among school children in Nigeria ranging from 1.2 to 6.9%^3–5^. Many conditions are known to cause vision impairment in children^6^, the leading cause identified as uncorrected refractive errors and amblyopia^6–8^.

Apart from vision, another important factor to consider is the environment in which students learn. Classroom size, gross area occupied by students, lettering size on learning materials as well as lighting are all major determinants influencing how students visualise information to learn^9,10^. A knowledge of the visual demand of school classrooms will further simplify appropriate classroom arrangements, to accommodate children with vision impairment.

Few studies^9–11^ have evaluated the visual demand in classrooms, however none have determined if children’s vision actually meets this demand. In addition, these studies have greater focus on primary schools. Simons^12^ has identified that interference with reading differs among the basic reading stages which are: the “learning to read” and the “reading to learn” stages. Children in nursery and early primary school, aged five to eight years, are grouped as those who are learning to read, from nine to 12 years they transition into the “reading to learn” stage while from age 13 to the age they finish school, it is expected that they become proficient at reading. At each stage, the visual demand may differ. Although it is argued that good vision in early school years is critical to academic performance, at the later stage of learning when more sustained effort is required as students spend more time reading to learn, it is important that the visual demand is also known.

In the US, Langford and Hug^11^ showed that the VA demand varies across elementary schools but, children with mild to moderate uncorrected refractive error and those with moderate to severe amblyopia, undergoing occlusion therapy, may do well in some classrooms if they are given seats in the centre of the front row. From their study, children in kindergarten to grade two had a distance VA demand ranging from 20/100 to 20/300 (LogMAR 0.7 to 1.2), while those in grades three to five had VA demand ranging from 20/60 to 20/100 (LogMAR 0.48 to 0.7). At near, all children had an average near VA demand that ranged from 20/100 to 20/500 (LogMAR 0.31 to 1.4). More recently, the study in India by Negiloni and colleagues^10^, among children in grade four to 12, showed that those with mild to moderate vision impairment can be accommodated in regular classrooms. The average distance VA demand at the furthest and closest sitting position were 0.31 and 0.56LogMAR, respectively. Average near VA demand was 0.44LogMAR. Also, a study of Australian primary classrooms^9^, which include computer usage, identified that high levels of VA, contrast and sustained accommodative convergence responses are required to perform well. The average VA demand reported was 0.33 and 0.72LogMAR at the furthest seating distance and at near, respectively. The average actual near demand, taking into consideration acuity reserve was 0.33LogMAR.

In the present study, we set out to determine for the first time if classrooms in Nigeria accommodate students’ VA needs. The measured habitual VA of students in primary and secondary schools were compared to the calculated VA demand values for their classrooms. It is hoped that results from this study will inform optometric management of visual conditions, as well as classroom functionality for all children in Nigerian classrooms.

## Methods

For this cross-sectional study, Schools in Oredo Local Government Area (LGA), one of the 18 LGAs in Edo State were sampled. Edo State is one of the 36 states in Nigeria; its capital is Benin City. Oredo LGA has 12 wards and occupies an area of 249 km^2^, housing a population of 374,671 as at the 2006 census (Wikipedia, April 2022). This LGA was chosen because of its location, spanning most of the city centre of Benin, and the ease of accessibility.

This study was conducted in accordance with World Medical Association Declaration of Helsinki on the ethical principles for medical research involving human subjects. Ethical clearance was obtained from the Edo State Ministry of Health ethical clearance committee. Approval was also granted from the Edo State Ministry of Education and, before study commenced, the head teacher or principal of each school. The study population comprised school children from Primary one to Senior Secondary School (SSS) three, attending registered Government (public) and private owned schools. The study was conducted from May to September 2018. At that time, there were 233 registered schools in Oredo LGA, comprising 14 public secondary schools, 88 public primary schools, 62 private secondary schools and 69 private primary schools. Using a list of schools obtained from the Ministry of Education, a total number of 35 schools were selected by systematic sampling as follows: the first school was randomly selected from the first ten on the list of schools thereafter, other schools were picked using the total number (233) divided by the sample size (35), as interval.

Each school agreed upon a convenient day to visit wherein no other major function was to take place. Using letters sent home, parents/guardians gave informed consent while verbal assent was sought from each child before their VA and reading distance were measured. Any student who had a general or ocular health condition that impeded evaluation, was excluded. At each school, the process of the research was explained to the head teacher/principal, classroom teachers and class students involved.

This study took place alongside another in which classroom activities were recorded, details of which are reported in a separate paper. Ethics approval allowed us access to only one class per year group (i.e. if, for example, Primary one had more than one classroom of students, we were allowed to evaluate only one of the classrooms), and this was assigned to us by the head teacher in such cases. Each school is expected to have six year groups i.e., either from primary one to primary six or from Junior Secondary School (JSS) one to SSS three. Given that our main aim was to compare the VA measured with the classroom VA demand, VA was only measured for students in those classrooms in which the VA demand was derived.

VA measurements were performed by the first author O.M.A. and five fifth- and sixth-year optometry students. These optometry students had considerable clinical experience of using the LogMAR chart in their course and the only training they needed was to provide consistency between recorders. Other trained research assistants took part in organizing the students when they were selected from their classrooms and escorted to the test space. VA was measured from at least two students in each classroom under observation. In classrooms with many students, students were chosen for testing by using their seat numbers to select every 5th student.

### VA measurement and near working distance

Before measurement began, the whole class was briefed on the test procedure and were assured that we were not going to touch their eyes during testing. Distance habitual VA (with spectacles if worn) was measured using a single Bailey-Lovie LogMAR acuity chart at four metres, in a room well-lit by daylight illumination. This chart is made up of 10 types British standard non-serifed letters, measuring 5 × 4 min of arc, and presented 5 per line with a uniform progression of 0.1 log units (starting from 0.8 assigned to the largest sized ones and − 0.5 to the smallest sized ones). In order words, each line has a value of 0.1LogMAR assigned to it while each letter has a value of 0.02LogMAR. LogMAR acuity charts have been identified to provide a more reliable VA measurement given that it provides between-letter and between-row spacings that are equivalent to the letter sizes, thereby balancing the level of difficulty for each row^13^.

During VA measurement, there was no randomization i.e. the right eye was always tested first, followed by the left and then both eyes, as it is done conventionally. The end point VA was determined using letter by letter scoring. In order words, the total LogMAR value for the line with the smallest letter(s) that the student could correctly identify. Where more than one letter was seen unsequentially on a line, each letter was assigned a value of 0.02LogMAR and the total subtracted from the preceding line, to arrive at the threshold value. Measurement was terminated when the school student made four or more mistakes on a line.

At 40 cm, we used the LEA symbol^®^ near vision card from Good-Lite vision. This multi-row chart has 5 × 5 matrix shapes of a house, apple, square and a circle and has scores for LogMAR units too. The chart design has 5 symbols included per row. The same specifications and recording methods as described with the distance measurement above were applied to the near testing. To determine habitual reading distance, ten randomly selected students from each class, were each given their notebook to hold and read while their near reading distances were measured and noted.

### VA demand

The VA demand values at distance and near were computed using procedure outlined in previous studies^9–11,14^. For this, the following information was required:

1. Smallest letter height in learning materials: This was measured using a millimeter scale and included textbook prints and randomly selected handwritings from students notebooks, consistent with a previous study^10^.
2. Distance at which the letter was viewed. This comprised the:
  - Furthest and nearest seating distance to the board
  - Average near working distance.

The VA demand in Snellen and thereafter, its LogMAR equivalent was calculated using the vertical height of letters and its viewing distance, with the assumption that the critical detail of each letter is one fifth of the letter height. A detailed explanation is given in the paper by Langford and Hug^11^. But as an example, assuming a letter W measuring 1.5inches (38 mm) in height is viewed from 110inches (2.79 m), the angular size of the letter in minutes of arc will be the arctan of the letter height divided by the viewing distance, giving us 46.74’. The Snellen’s equivalent can then be derived by dividing the size of the letter in minutes of arc by 5 and multiplying by 6; assuming that a letter E, for example, at 6/6 will have each light and dark bar subtending at 1 min of arc thereby giving a total of 5 min of arc. The letter W then produces a VA demand of 6/56 (20/187), in Snellen. Furthermore, The log of the reciprocal of 6/56 will give the equivalent VA demand of 0.97LogMAR. The measured viewing distance allowed calculation of: minimum distance VA demand (nearest seat), maximum distance VA demand (furthest seat) and near VA demand.

The *actual* near VA demand was further derived. Studies have found that for children to read fluently and comfortably, an acuity reserve (i.e. print size relative to near acuity) of 2.5 times the threshold VA is required^15,16^. This is because, the demand provided by the existing near reading material is less by a factor of 2.5× the acuity needed. Consistent with previous study^9^, the *actual* near VA demand was thereafter obtained by subtracting 0.398LogMAR (given that a 2 × acuity reserve is equivalent to 0.3 log units or 3 LogMAR lines, and a 3 × acuity reserve is equivalent to 0.5 log unit or 5 LogMAR lines^17^) from the near VA demand, to account for this acuity reserve.

For comparison with measured habitual VA, the VA demand at maximum distance was used. This is because it is expected that the furthest seating position would pose more difficulty while viewing the smallest letters.

### Data analysis

This was done using the R statistical software version 3.5 and the Statistical Package for Social Science (SPSS) version 23. At 0.05 significance level, the t-test was used to determine differences between measured VA and calculated VA demand. Pearson’s correlation coefficient was used to examine the association between age of students and the VA demand at distance and near.

## Results

Among the 35 schools approached, three private owned schools declined, while for seven others, results were not obtained because of the Nigerian Labour Congress (NLC) strike action, and the postponement of resumption of all public schools by the Edo State Government. Therefore, a total of 25 schools were visited, comprising: seven public primary schools and six each of public secondary, private owned primary and private secondary schools. Data were obtained from 148 classrooms, as two private-owned primary schools had no students in one class.

### Measured habitual VA values at distance and near

Visual acuity was measured in 2,234 children. The highest number of VA results obtained for a class was 31 while the lowest was two. Mean measured habitual VA is shown in Table 1.

**Table 1:** Measured habitual LogMAR VA for all students.

	Distance VA			Near VA		
	RE	LE	Binocular	RE	LE	Binocular
Mean	− 0.06	− 0.068	− 0.12	− 0.06	− 0.067	− 0.10
Median	− 0.10	− 0.10	− 0.10	− 0.10	− 0.10	− 0.10
Standard deviation	0.18	0.17	0.17	0.15	0.15	0.15
Minimum	− 0.5	− 0.40	− 0.50	− 0.3	− 0.3	− 0.3
Maximum	1.58	1.58	1.58	1.20	1.20	1.20

*RE* Right eye, *LE* Left eye.

**Figure 1:**
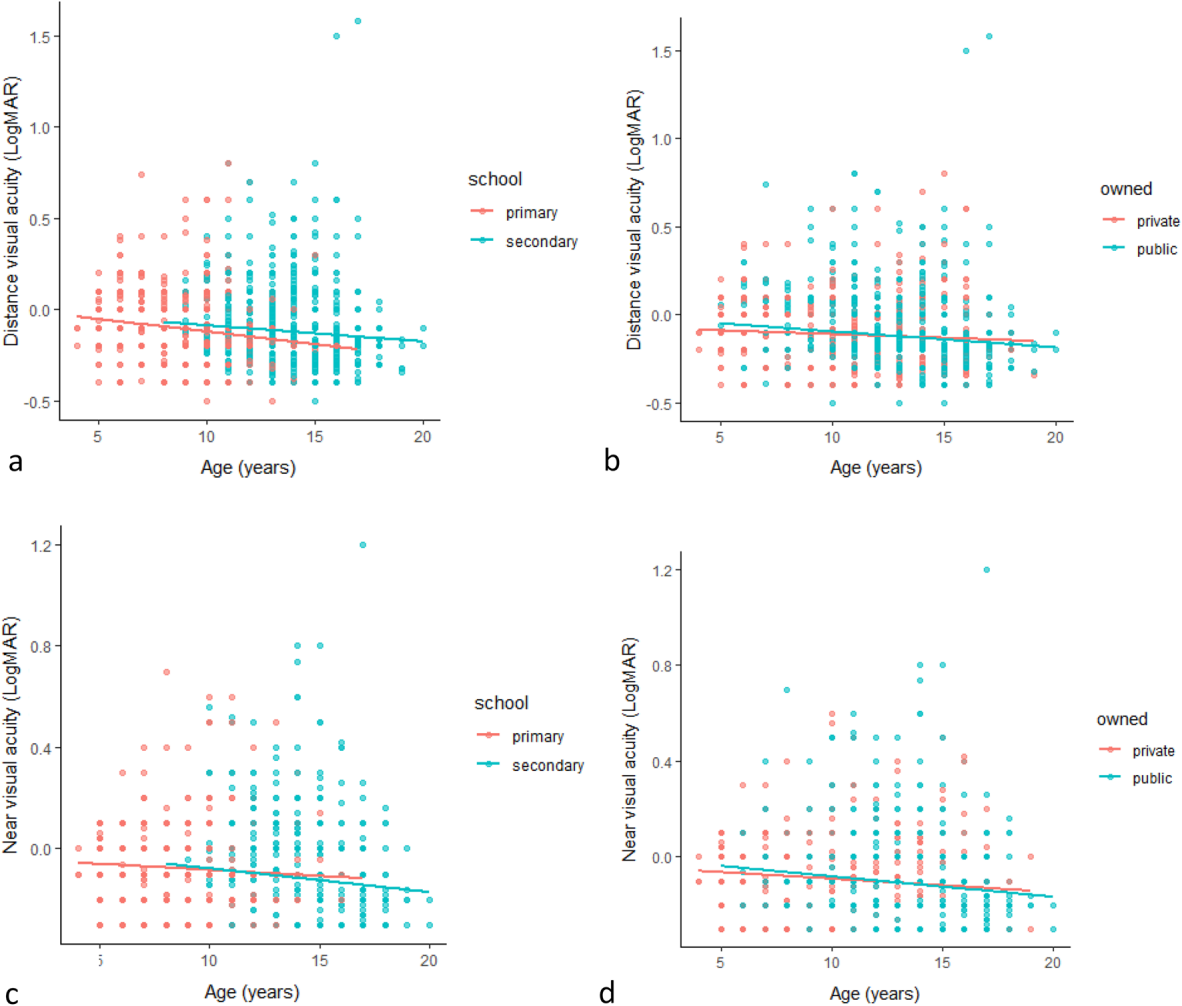
Distance and near VA as a function of age; classified into school types (**a** and **c**) and school ownership (**b** and **d**).

The distance and near measured VA is also represented, as a function of age, in Fig. 1. Results show that there is a significant negative correlation between age and VA in primary [*n* = 947; distance VA (r =  − 0.208, *p* = 0.000); near VA (r =  − 0.085, *p* = 0.009)], secondary [*n* = 1287; distance VA (r =  − 0.102, *p* = 0.000); near VA (r =  − 0.122, *p* = 0.000)], public [*n* = 1,421; distance VA (r =  − 0.149, *p* = 0.000); near VA (r =  − 0.159, *p* = 0.000)] and private [*n* = 813; distance VA (r =  − 0.083, *p* = 0.018); near VA (r =  − 0.130, *p* = 0.000)] schools. In other words, VA measured (decreased in LogMAR values) improved with increasing age.

Using the World Health Organisation (WHO) classification of vision impairment, students habitual VAs were also categorised, see Table 2. All subsequent statistical analysis was conducted using binocular VA.

**Table 2:** Classification of measured habitual VA among all students.

Terminology	WHO classification (LogMAR)	Distance VA number (%)			Near VA number (%)		
		RE	LE	Binocular	RE	LE	Binocular
Mild or no VI	≤ 0.50	2201 (98.50)	2208 (98.80)	2216 (99.20)	2217 (99.20)	2217 (99.20)	2223 (99.51)
Moderate VI	0.52–1.0	31 (1.40)	24 (1.10)	16 (0.70)	15 (0.70)	15 (0.70)	10 (0.45)
Severe VI	1.02–1.30	–	–	–	2 (0.10)	2 (0.10)	1 (0.04)
Blindness	1.32–1.80	2 (0.10)	2 (0.10)	2 (0.10)	–	–	–
Total		2234	2234	2234	2234	2234	2234

*RE* Right eye, *LE* Left eye, *VI* Vision impairment.

### Calculated VA demand at distance and near

**Figure 2:**
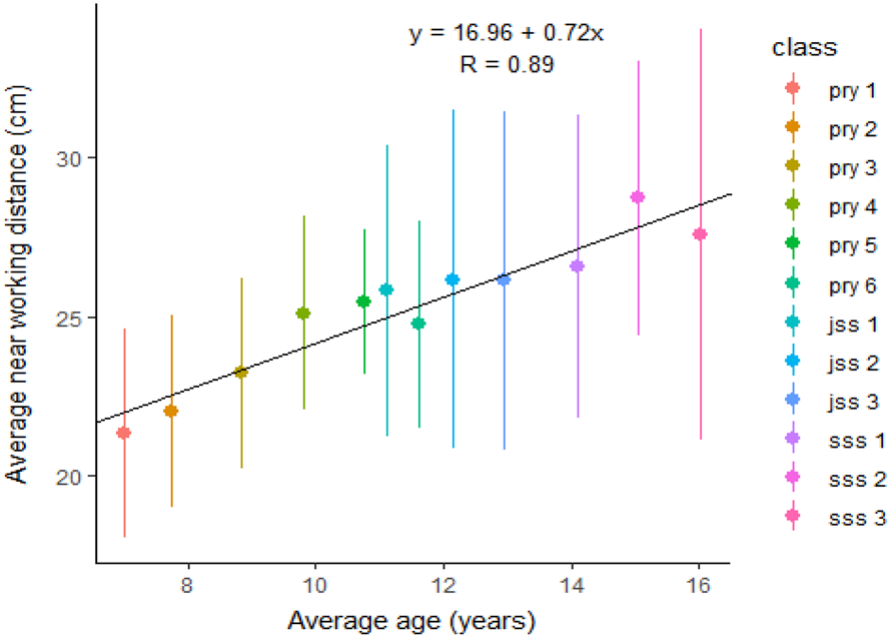
The relationship between average near working distance and age of students.

The distance and near VA demand in 148 classrooms, actual near VA demand, as well as parameters used to calculate them are presented in Table 3. Figure 2 further illustrates the relationship between average near working distance and age of students, showing that as age increases average near working distance increased. The difference in VA demand values, obtained using the unpaired t-test, is represented in Table 4.

**Table 3:** Calculated VA demand across all schools (148 classrooms) and parameters used in calculation.

	Mean	Median	SD	Range	
				Min	Max
Distance from board to closest seat (m)	2.52	2.33	0.99	1.11	8.80
Distance from board to furthest seat (m)	6.63	6.75	1.92	2.10	11.50
Average near working distance (cm)	25.18	25.00	4.53	15.00	37.20
Smallest letter height viewed at near (mm)	1.50	1.50	0.50	1.00	3.00
Smallest letter height viewed at far (mm)	14.69	15.00	3.99	7.00	28.00
VA demand at minimum distance (LogMAR)	0.65	0.62	0.33	0.26	3.97
VA demand at maximum distance (LogMAR)	0.21	0.18	0.23	− 0.13	2.20
Near VA demand (LogMAR)	0.60	0.60	0.17	0.14	1.00
Actual near VA demand (LogMAR)	0.20	0.21	1.70	− 0.26	0.62

*SD* Standard deviation.

**Table 4:** Difference in VA demand values.

		Mean (Snellen equivalent and font size at 40 cm, included in bracket)	SD	SEM	*p*-value	Direction of difference
**Between public (n = 78) and private (n = 70) school classrooms**						
VA demand (LogMAR) at minimum distance	Public	0.65 (6/26.8)	0.41	0.05	0.950	n/a
	Private	0.65 (6/26.8)	0.19	0.02		
VA demand (LogMAR) at maximum distance	Public	0.15 (6/8.5)	0.26	0.03	**0.001**	Public classrooms had more VA demand. They had longer average distance to furthest seats (8.00 vs 5.10 m)
	Private	0.27 (6/11.2)	0.18	0.02		
Actual near VA demand (LogMAR)	Public	0.17 (6/8.9 or N4.7)	0.13	0.01	**0.021**	Public classrooms had more VA demand. The average letter height measured at near was smaller for these classrooms (1.31 vs 1.72 cm)
	Private	0.24 (6/10.4 or N5.5)	0.21	0.02		
**Between primary (n = 76) and secondary (n = 72) school classrooms**						
VA demand (LogMAR) at minimum distance	Primary	0.66 (6/27.4)	0.43	0.05	0.584	n/a
	Secondary	0.63 (6/25.6)	0.16	0.02		
VA demand (LogMAR) at maximum distance	Primary	0.24 (6/10.4)	0.29	0.03	**0.035**	Secondary school classrooms had more VA demand. They had slightly longer average distance to furthest seat (6.68 vs 6.57 m)
	Secondary	0.16 (6/8.7)	0.14	0.02		
Actual near VA demand (LogMAR)	Primary	0.31 (6/12.3 or N6.6)	0.13	0.02	** < 0.0001**	Secondary school classrooms had more VA demand. These classrooms had smaller average letter height in reading materials (1.19 vs 1.80 cm)
	Secondary	0.09 (6/7.4 or N4)	0.12	0.01		

Significant values are in [bold].

*SD* Standard deviation, *SEM* Standard error of mean.

### The VA demand in each class level as a function of age

**Figure 3:**
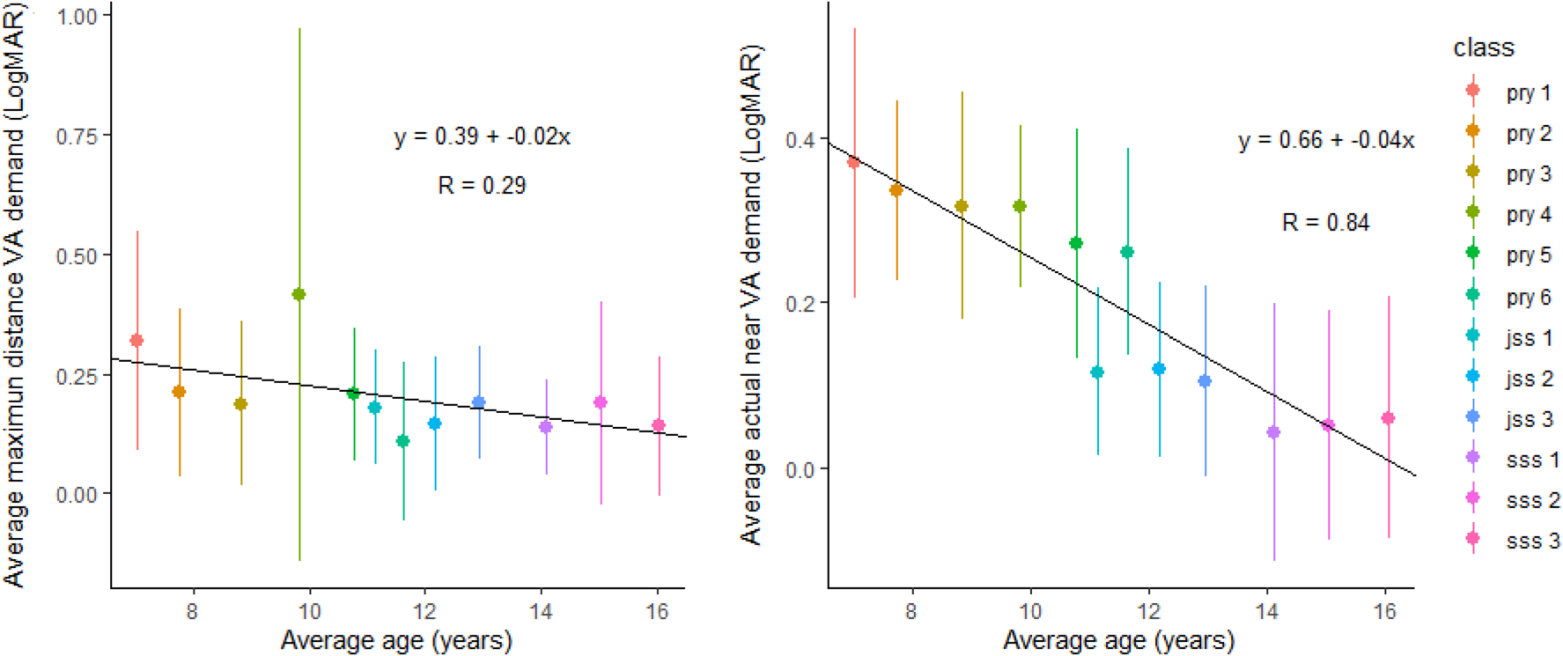
Average maximum distance VA demand (left) and average actual VA demand (right), as it relates to the average age of students per class.

Using the average age for each class i.e., primary one to SSS3 and the maximum distance VA demand, as well as the average actual near VA demand, the relationship between age and VA demand was determined using Pearson’s Correlation. As shown in the scatter plots in Fig. 3, an increase in age relates to a decrease in LogMAR VA demand values at both distance (r =  − 0.549, *p* = 0.070) and near (r =  − 0.921, *p* < 0.0001). This translates to an increase in task demand with increasing age.

### Difference between the measured VA and the calculated VA demand

Using the one-sample t-test, the measured VA at distance and near was compared with the VA demand at the maximum distance and the actual near VA demand, respectively. In primary and secondary schools, at distance and near, the measured VA was significantly better than the calculated VA demand (*p* < 0.0001).

Among all 947 students in Primary schools, 97.78% had VA better or equal to the average maximum distance VA demand for primary school classrooms [0.24LogMAR (6/10.4)], while 98.83% of students had VA better than or equal to the average actual demand at near [0.31LogMAR (6/12.3 or N6.6 at 40 cm)]. On the other hand, among all 1287 students in secondary schools, 94.09% had VA better or equal to the average maximum distance VA demand for secondary school classrooms [0.16LogMAR (6/8.7)], and 90.60% of students had VA better than or equal to the average actual demand at near [0.09LogMAR (6/7.4 or N4 at 40 cm)].

The percentage number of students whose VA was poorer than the calculated VA demand, at distance and near, was determined for each class (primary one to SSS3). This is represented in Table 5.

**Table 5:** The percentage number of students in each class with VA poorer than the mean VA demand at distance and near.

Class	Mean age (years)	Mean maximum distance VA demand (LogMAR)	Mean actual near VA demand (LogMAR)	VA poorer than mean maximum distance VA demand (%)	VA poorer than mean actual near VA demand (%)	Average number of students per class
Primary 1	7.02	0.32	0.38	1.22	0.61	34.92
Primary 2	7.74	0.21	0.33	1.24	0.62	38.62
Primary 3	8.84	0.19	0.32	2.75	0.55	36.31
Primary 4	9.83	0.41	0.32	1.34	2.01	32.00
Primary 5	10.77	0.21	0.27	3.90	2.60	30.67
Primary 6	11.63	0.11	0.26	2.19	2.19	34.67
JSS 1	11.13	0.18	0.12	5.05	5.50	59.67
JSS 2	12.16	0.14	0.12	4.85	3.52	66.75
JSS 3	12.95	0.19	0.11	6.28	6.28	58.25
SSS 1	14.10	0.14	0.04	4.90	9.31	49.58
SSS 2	15.05	0.19	0.05	7.79	10.82	42.69
SSS 3	16.03	0.14	0.06	7.07	7.61	30.09

*JSS* Junior Secondary School, *SSS* Senior Secondary School.

## Discussion

This study evaluated students’ habitual VA, and provides novel findings of VA demand from Nigerian classrooms, as well as measured VA of students compared with the calculated VA demand for their classrooms. For the majority of children, measured VAs were better than the calculated VA demand. However, up to 9.40% of children overall, did not have good enough VA to meet the classroom demands. Almost 1% of students had VI or blindness at distance, near or both, using WHO classifications. A previous study among 1144 children in Nigeria showed that 1.48% had VI or blindness at distance, of which the majority resulted from uncorrected refractive error^3^.

Values for maximum distance VA demand obtained from this study, were similar to findings from previous studies^9,10^, which ranged from about 0.24 to 0.35LogMAR. Contrary to these findings, one recent study by Adams and colleagues^14^ which evaluated 12 kindergarten to grade 12 classrooms, in New York city, found mean maximum VA demand value of 0.42LogMAR. The classroom sizes from their study measured a mean of 18ft (5.5 m) in length, hence the furthest seating distance will have been closer than in the classrooms evaluated in our study and in previous papers. Again, further buttressing the effect of classroom size, Adams et al.^14^ reported that minimum and maximum distance VA demand did not differ significantly between grade levels. This conflicting results may have also resulted from the difference in the number of classrooms evaluated for both studies. In the current study, the VA demand at maximum distance, and the actual near VA demand differed significantly between public and private schools, and between primary and secondary schools (Table 4). The private school classrooms as well as the primary school classrooms put less demand on the visual system, which may have resulted from smaller classroom sizes in the private schools. The classroom size, distance to the board as well as lettering size on the board, are important parameters needed to derive the visual demand for a particular learning space. Smaller classrooms with larger letter sizes on the board, produce lower demands on the visual system and hence are better for students with poorer vision. At near, the measured habitual near working distance varied; younger children in primary schools had shorter near working distances (Fig. 2) and larger letter size in materials, explaining the significant difference in actual near VA demand between primary and secondary. Interestingly, we found that public schools had lower average letter height in materials available for near viewing. This finding may be purely coincidence or could have occurred because children in public schools wrote in smaller sizes to minimize writing space, considering the link between wealth and private school education^18^.

From this study, the average near working distance measured (Table 3) was 25.18 cm (ranging from 15.00 to 37.20 cm); this means an accommodative demand of 2.70 to 6.67D. More so, it was interesting to find near working distances shorter than is generally accepted clinically. Clinical procedures are conducted at a typical working distance of 40 cm, whereas, as shown here, children use a much closer distance. Our average near working distance of 25.18 cm was similar to that found in previous study, in which children from kindergarten to year 6 had an average near working distance of 10inches (25.40 cm)^19^.

The scatter plots in Fig. 3 further indicate that with increasing age there is an increase in task demand on the visual system. This finding was more prominent at near, as the near VA demand increased (size of print decreases) significantly in older children. From our study, the furthest seating distances to board in secondary school classrooms were longer, and these children also had a longer near working distance (Fig. 2). In addition, the teachers were often seen to put much more information on the boards for secondary school children than in primary schools therefore, letter sizes on writing boards were smaller. Findings also indicate that letter sizes in reading materials (especially formulae in maths textbooks) were smaller. These may have accounted for the increasing number of students in secondary schools with VA poorer than their classroom demands.

Table 5 also showed that more students in secondary schools had measured habitual VA poorer than the maximum distance VA demand. One explanation for this finding could be the increasing prevalence of myopia with age^20^. Often children who are emmetropic on entering school, become myopic later. This common form of myopia develops at about age 6 and progresses through 20 years^21^. This study has limitations in this aspect as the cause of reduced vision was not investigated since it was beyond the scope of this study.

Some other limitations of this study include first, we assumed a general height to stroke width of 5:1 for all letters, not taking into consideration that the corresponding acuity threshold demand may differ significantly with the actual stroke width measurement. This was necessary to avoid complications while deriving the results which may arise from different board types, writing material used and letter sizes encountered in the classrooms evaluated. Second, the VA measurement of right before left in all cases may have resulted in increased familiarity with the task and the letters presented thereby translating to better VAs obtained for the left eye. Third, the effect of luminance on visual performance was not explored therefore information about the level of luminance on visual tasks and how this relates to visual performance cannot be inferred from this study. Lastly, electronic device are becoming increasingly useful for near tasks because they have an advantage of modifying contrast and size to meet individual needs, however since no electronic device for learning were encountered in the classrooms visited, letter sizes in these devices were not measured, hence our findings cannot be generalized to accommodate this.

In computing the data for this paper, we chose to use the closest and furthest viewing distances for the distant visual task, to give an overall picture of the range of demand the classrooms gives i.e. showing both extreme values. This we believe can easily be translated to practical solutions needed to enhance distance vision in the classroom environment, unlike for near viewing. Hence, the use of the average near working distance taken from a sample of students rather than the maximum or minimum. For a student to be able to see all information on the board at the furthest seating distance in their classroom, they will need to have VA equal to or better than the maximum VA demand. Similarly, for comfortable reading, a student will need near VA equal to or better than the actual near VA demand. Therefore, VA demands represent the cut-off point in which a child may begin to have problems in the classroom. Children whose vision is poorer than the demand may be able to cope if adjustments are made, such as seating closer to the board, or text on reading materials being enlarged.

The majority of students in this study had better VA than the classroom demands, however for the few who did not, it is crucial that teachers understand the issues and accommodate them. As part of the Edo-BEST programme, teachers in public primary schools one to three, were trained to measure students’ distance VA. Using the Snellen’s chart, students with VA poorer than 6/12 were positioned in a front seat. No secondary and private schools had such system in place yet. This system however works on the assumption that the child may have myopia or amblyopia and would benefit from a closer viewing distance. Other cases in which a student has astigmatism or severe accommodative disorders impacting near vision may often go unnoticed, and for these comprehensive eye care intervention will also be required.

Inclusive education seems to be the ongoing trend in childhood education. More needs to be done to accommodate children with reduced vision in regular classrooms. To achieve this aim, adequate classroom environment, teacher support and training as well as the required visual aids are crucial^22,23^. Individualized learning and the deployment of strategies to improve social interaction with other students is important to promote learning for the visually impaired^22^.

In summary, considering the distance VA demand values and the actual near VA demand (taking into account acuity reserves), a child whose habitual distance VA is poorer than 0.21LogMAR (6/9.6 or 20/32) but better than 0.65LogMAR (6/26.6 or 20/89) may view all content of the board in classrooms in Oredo LGA. Students with a habitual near VA poorer than 0.20LogMAR (6/9.5 or 20/32 or N12.8 at 40 cm), may find it difficult to read comfortably the smallest letters in materials provided. Most students had VA better than their classroom demands. However, up to about 11% of school children had VA poorer than their classroom demands and needed full eye examinations. Those children whose vision cannot be improved will benefit from a closer seating position to the board or larger letterings/numbers in reading materials.

Based on our findings, we therefore recommend that early detection and intervention for students with vision impairment and other vision defects should be encouraged. More teachers and private school owners should be educated on the need to, and taught how to, identify children who have visual problems for prompt referral, and who may benefit from classroom modifications to subsequently influence classroom performance. In this light, teachers should be trained on how to adapt their board work while textbook prints be made available with larger font sizes. Perhaps, if teachers wrote large and legible letters on the board and if schoolbooks were written with larger font sizes, more students would access distance targets and reading will be easier. The VA demand in classrooms would be less, and more students with uncorrectable vision impairment would be accommodated. In addition, we also recommend that other factors necessary for a holistic visual experience be considered for future studies. Most importantly, the contrast demand of target materials.

This study has provided original information on the VA and VA demand in primary and secondary school classrooms in Oredo LGA. Results are expected to guide vision screening protocols and referral criteria for school children in this area. It may also serve as a guideline to accommodate students with vision impairment in regular school classrooms. In addition, this study has added to the body of existing knowledge on the magnitude of vision impairment and blindness present among school children in Oredo LGA.

## Data Availability

The datasets generated during and/or analysed during the current study are available from the corresponding author on reasonable request.
